# Phenotypic and Genotypic Analysis of Resistant *Helicobacter pylori* Strains Isolated from Children with Gastrointestinal Diseases

**DOI:** 10.3390/diagnostics10100759

**Published:** 2020-09-27

**Authors:** Monika Maria Biernat, Aldona Bińkowska, Łukasz Łaczmański, Paweł Biernat, Paweł Krzyżek, Grażyna Gościniak

**Affiliations:** 1 Department and Clinic of Haematology, Blood Neoplasms, and Bone Marrow Transplantation, Wroclaw Medical University, 50-367 Wroclaw, Poland; monika.biernat@umed.wroc.pl; 2 2nd Military Field Hospital of the Polish Armed Forces, 50-984 Wroclaw, Poland; aldona.binkowska@gmail.com; 3 Hirszfeld Institute of Immunology and Experimental Therapy, Polish Academy of Sciences, 53-114 Wroclaw, Poland; lukasz@diagmol.com; 4 Department of Drugs Form Technology, Wroclaw Medical University, 50-556 Wroclaw, Poland; pawel.biernat@umed.wroc.pl; 5 Department of Microbiology, Wroclaw Medical University, 50-368 Wroclaw, Poland; grazyna.gosciniak@umed.wroc.pl

**Keywords:** *Helicobacter pylori*, children, antibiotic susceptibility, clarithromycin, metronidazole, point mutations

## Abstract

Antibiotic resistance of *Helicobacter pylori* is currently a global issue. The aim of this study was to analyze actual antibiotic resistance rates of *H. pylori* strains isolated from children with primary infections and to compare the incidence of mutations that determine resistance to clarithromycin (CH) and metronidazole (MET) in children with different clinical diagnoses. A total of 91 *H. pylori* strains were isolated from 108 children with primary infections. Drug susceptibility testing of the strains was performed using E-test method. Classical sequencing of DNA fragments was used to detect point mutations for CH and MET resistance. Resistance to CH was detected in 31% of isolated strains (28/91), while resistance to MET and CH was detected in 35% (32/91) of strains. A2143G was the most frequently detected mutation and was dominant among strains isolated from children with peptic ulcer disease (80%). Mutations in the *rdxA* gene were found significantly more frequently among MET-resistant strains than MET-sensitive strains (*p* = 0.03, Chi^2^ = 4.3909). In children, a higher frequency of *H. pylori* multiresistant strains was observed compared with the previous study in the same area. Differences were found in the occurrence of point mutations among *H. pylori* strains resistant to CH isolated from children with different clinical diagnoses.

## 1. Introduction

Infections caused by *Helicobacter pylori* occur frequently in children. In most cases they are asymptomatic, but in a small percentage of infected children they may lead to peptic ulcer disease, erosive gastritis or duodenitis, which are all associated with abdominal pain and dyspepsia. Extragastric complications, such as iron-deficiency anemia and immune thrombocytopenic purpura, may also occur [1,2]. Serious complications—including gastric cancer or mucosa-associated lymphoid tissue (MALT) lymphoma—may occur many years after primary infection and are associated with a chronic inflammation process that leads to angiogenesis and pathologic changes in the gastric mucosa [3,4]. The association between *H. pylori* infection and gastroesophageal reflux disease (GERD) or inflammatory bowel disease (IBD) is controversial. Recent studies have shown that the frequency of *H. pylori* gastritis in children with IBD is lower than that in control individuals [5]. *H. pylori* infection may exacerbate GERD but may also have a protective role. An inverse relationship between the grade of esophagitis and the presence of *H. pylori* has been reported, which also shows that the presence of *H. pylori* seems to be beneficial and that it does not increase the grade of esophagitis [6,7].

Infections produced by *H. pylori* are only treated in symptomatic children and the eradication is based on complex antibiotic therapy according to the European Society of Pediatric Gastroenterology Hepatology and Nutrition (ESPGHAN)/North American Society for Pediatric Gastroenterology, Hepatology and Nutrition (NASPGHAN) guidelines [8]. In first-line therapy, protein-pump inhibitors (PPIs) and antibiotics from the macrolide group, mainly clarithromycin (CH), are used in combination with amoxicillin (AMO) and nitroimidazole, including mainly metronidazole (MET) and less frequently tinidazole [8].

Failures of *H. pylori* infection therapy are mainly caused by increased antibiotic resistance, predominately to CH. *H. pylori* resistance is widespread, and the frequency of its occurrence varies regionally. Resistance to CH results from point mutations in the domain V of the 23S rRNA gene of the 50S ribosomal bacterial subunit, which lead to changes in protein conformation [9]. Three point mutations are of the greatest clinical importance: A2143G, A2142G and A2142C. The frequency of mutations varies depending on the geographic region. In the United States and Europe, the A2143G mutation is most frequently detected in strains resistant to CH [10]. Resistance to MET is mainly caused by mutations in the *rdxA* gene, which encodes the oxygen insensitive RdxA nitroreductase. Mutations in the *rdxA* gene may lead to a lack of enzymatic activity in this protein [11,12].

The aim of this study was to analyze the actual antibiotic resistance rates of *H. pylori* strains isolated from children with primary infections and to compare the incidence of mutations determining the resistance to CH and MET in children with different gastrointestinal diseases.

## 2. Materials and Methods

### 2.1. Clinical Data

A retrospective study was carried out on 91 *H. pylori* strains isolated from 108 children with primary infection, who were diagnosed and treated at the 2nd Department and Clinic of Pediatrics, Gastroenterology and Nutrition of the Wroclaw Medical University from April 2016 to March 2019. The strains were isolated from biopsy samples of gastric mucosa tissue, which were taken during endoscopic examinations of the upper gastrointestinal tract performed for clinical indications. Children with symptoms of nausea and vomiting, abdominal pain and gastrointestinal bleeding, in whom the infection had not been previously eradicated, were analyzed. The exclusion criteria were a history of previous *H. pylori* infection, antibiotic use, non-steroidal anti-inflammatory drug use or PPI use in the four weeks preceding the study. In children involved in the study, after obtaining the written consent of the parents, an endoscopic examination was performed to obtain both the gastric antrum and corpus for histopathological analysis (according to the updated Sydney classification). The approval of the Bioethics Committee of Wroclaw Medical University was obtained for the study (No 111/2017; 25 April 2017). The diagnosis of gastrointestinal disease was based on clinical, endoscopic and histologic findings. The diagnosis of *H. pylori* infection was based on histology and the culture of biopsy specimens. The bacteriological examination of gastric mucosa fragments taken during endoscopic examination was based on direct Gram staining, culture and biochemical differentiation (urease, oxidase and catalase) [13]. The reference strain, *H. pylori* J99, from the collection of the Department of Microbiology of Wroclaw Medical University was used in this study.

Initially, the biopsy samples collected during endoscopy for microbiology examination were placed in sterile saline (0.15 M NaCl) and processed within two hours. Then, biopsy samples were placed on two culture media: Columbia agar (Difco, Lublin, Poland) with 7% hemolyzed horse blood and selective medium Columbia agar (Difco, Lublin, Poland) with 7% hemolyzed horse blood enriched with a selective supplement (Oxoid, Le Pont de Claix, France). The supplement contained vancomycin 10 mg/L, trimethoprim 10 mg/L, cefsulodine 5 mg/L, and amphotericin B 5 mg/L. Incubation was performed under microaerophilic conditions (10% CO_2_, 85% N_2_, 5% O_2_) at 37 °C for 3–5 days. Isolated strains were identified on the basis of characteristic morphology of colonies and biochemical properties such as the ability to produce catalase, oxidase and urease. The epsilometer test (E-test) method was performed, as previously described [14,15]. *H. pylori* isolates were considered resistant when the minimum inhibitory concentration (MIC; μg/mL) was CH > 0.5, levofloxacin (LEV) > 1, tetracycline (TET) > 1, MET > 8 and AMO > 0.12 [13].

### 2.2. Detection of H. pylori Genomic DNA Fragments

Detection of *H. pylori* genomic DNA fragments by PCR using a previously described method [16]. DNA was isolated from *H. pylori* strains after 72 h of incubation with the column method using a Genomic Mini KIT (A&A Biotechnology, Gdynia, Poland). We chose strains that were passaged on culture media only two or three times to reduce the risk of the high genetic variability of analyzed regions. After the centrifugation and removing the supernatant, the initial stage of cell lysis was initiated. The resulting suspension was transferred to columns and centrifuged for 1 min at 11,000× *g*. Then, the filtrate was discarded, 500 µL of washing buffer was added and centrifuged for 1 min at 11,000× *g*. Again, the filtrate was discarded, 600 µL of washing buffer was added and centrifuged for 1 min at 11,000× *g*. After discarding the filtrate to remove ethanol residues from the column, the samples were centrifuged at 11,000× *g* for 1 min and moved to an Eppendorf column. The final step was DNA elution from the column. After the addition of 100 µL of the elution buffer heated to 70 °C and a one minute incubation at room temperature, samples were centrifuged for 1 min at 11,000× *g* to obtain pure *H. pylori* DNA. The isolated DNA was stored at −20 °C. polymerase chain reaction (PCR) for all strains was carried out on an MJ Research PTC-200 thermocycler. Complementary oligonucleotides to individual fragments of genomic DNA from *H. pylori* from Generi Biotech (Czech Republic) and PCR polymerase TaKaRa TaqTM hot start version from TAKARA Co (Japan) were used. The composition of the reaction mixture for the 23S rRNA *H. pylori* fragment and reaction conditions were described elsewhere [13]. The reactions were carried out in a final volume of 20 µL/sample. The size of the obtained product was 270 bp. The primers for the *rdxA* gene were as follows: forward 5′–3′: GCA GGA GCA TCA GAT AGT TCT, reverse: 5′–3′: GGG ATT TTA TTG TAT GCT ACA A. The PCR conditions for *rdxA* gene fragments were as follows: 10 min at 94 °C for initial denaturation, followed by 35 cycles of 40 s at 94 °C, 40 s at 50 °C and 1 min at 72 °C, with a final round of 10 min at 72 °C. The size of the *rdxA* product obtained was 886 bp.

### 2.3. Classical Sequencing of H. pylori DNA Fragments

Classical sequencing of *H. pylori* DNA fragments by PCR as described elsewhere [16]. Briefly, in the first step, products were purified from the excess of ssDNA/oligonucleotides and dNTPs. Final products were subjected to classical sequencing with a BigDye^®^ Terminator v3.1 cycle sequencing kit (Applied Biosystems, Foster City, CA, USA). The following were added to each sample: 0.5 µL starter forward, 2.5 µL BigDye, 2 µL BigDye buffer and 9 µL water. The reaction was carried out under the following conditions: 96 °C for 1 min, then 35 cycles of 96 °C for 10 s, 50 °C for 5 s and 60 °C for 5 min. After the reaction was complete, the products were purified again from unbound reagents using FastAP alkaline thermosensitive phosphatase under the same conditions. A total of 5 µL of the product and 9 µL of formamide (Hi-Di™; Applied Biosystems, Foster City, CA, USA) were collected for separation on a denaturing polyacrylamide gel in a 3700 DNA analyzer sequencer. The results were analyzed with the FinchTV program. Sequenced DNA fragments of *H. pylori* strains were analyzed using the free FinchTV Version 1.5.0 application developed by the Geospiza research team. Each sequence was compared to the sequence of the *H. pylori* J99 and *H. pylori* 26695 reference strains, whose entire genomes are available on the GenBank website.

### 2.4. Statistical Analysis

Statistical analyses were based on the assigned multi-divisional quantity tables and included comparisons within the groups (resistant–sensitive/male–female). First, general correlations between analyzed variables were assessed using correspondence analysis and the generalized analysis of main components. Statistical significance between nominal variables was evaluated by the chi-quadrant test with Yates’ correction and the exact Fisher’s test. The *p* value of ≤0.05 was considered statistically significant. All data were analyzed by STATISTICA v. 10.0 (StatSoft, Tulsa, OK, USA).

## 3. Results

**Figure 1:**
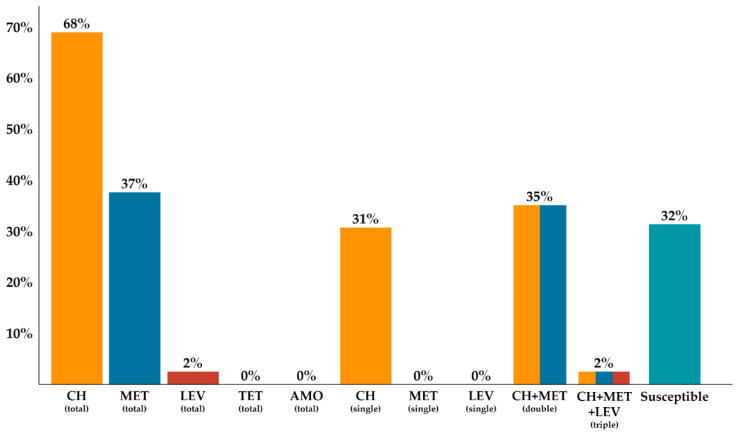
Resistance of *Helicobacter pylori* strains isolated from children with primary infections.

In this study, we examined 91 *H. pylori* strains, which were isolated from gastric mucosa biopsy samples from 108 children (56 girls and 52 boys) with various gastrointestinal diseases (2–18 years, median 12.5 years). Of all tested *H. pylori* strains, 38.5% (35/91) were isolated from patients with chronic gastritis, 15% (14/91) from patients with GERD, 16.5% (15/91) from children with gastric and/or duodenal ulcer disease and 27/91 (30%) from patients with other gastrointestinal diseases, such as coeliac disease (5/27), IBD (7/27), iron deficiency anemia (6/27), growth retardation (4/27) or allergies (5/27) (Table 1). Antrum-predominant gastritis was observed in GERD patients, whereas in patients with IBD the most frequent endoscopic diagnosis was gastroesophagitis. Among the 91 *H. pylori* strains isolated from children with primary infection, 32% (29/91) were sensitive and 68% (62/91) were resistant to at least one of the tested antibiotics. Among tested strains, 31% of strains (28/91) were resistant to CH, 35% (32/91) were resistant to MET and CH and 2% (2/91) were resistant to MET, CH and LEV. Strains resistant only to MET were not detected. All examined strains were sensitive to AMO and TET (Figure 1). CH-resistant strains were detected significantly more frequently in girls than boys (*p* = 0.032). Moreover, these strains were isolated more often than sensitive strains in patients with gastric/duodenal ulcers and GERD (*p* = 0.03 and *p* = 0.01, respectively). Multiresistant strains, including those resistant to CH and MET, were detected significantly more frequently in boys with peptic ulcer disease and chronic gastritis *(p <* 0.05, Table 1).

The mutation analysis of the 23S rRNA gene was performed on 62 *H. pylori* strains resistant to CH and 29 sensitive to CH. We chose strains that were passaged on culture media only two or three times to reduce the risk of the high genetic variability of analyzed regions [17]. Point mutations were detected in 55/62 resistant strains. Among them, 61% (38/62) had the A2143G mutation, 44% (27/62) had a single mutation, and the others had at least two mutations. Among strains resistant to CH, 11% (7/62) had the A2142G mutation, of which six strains had a single mutation and one strain had at least two mutations. In the remaining 10% (6/62) of resistant strains, no mutation was found. Among strains sensitive to CH, 7% (2/29) had the A2143G mutation, of which 3% (1/29) had A2143G + A2174G mutations. Moreover, 17% (5/29) of strains had T2182C mutations. The remaining 72% (21/29) of the sensitive strains had no mutations in the 23S rRNA gene. Differences in detected point mutations were observed among strains isolated from children with different diseases. The CH-resistant strains with the A2143G mutation were isolated most often, regardless of the patient group (Table 2). In the group of patients with peptic ulcer disease, these strains constituted as much as 80% of all resistant strains, whereas in the group of patients with other diseases and GERD, they constituted approximately 40% of resistant strains; however, the differences were not statistically significant. Strains resistant to CH that had no nucleotide changes in the examined fragment of the 23S rRNA gene and sensitive strains were nearly absent in patients with gastric and/or duodenal ulcer disease. In the group of patients with other gastrointestinal diseases, including IBD, coeliac disease and allergies, sensitive strains without mutations in the examined gene fragment were often present (33%, 9/27). Strains resistant to CH with the A2142G mutation were most often present in patients with GERD (21%, 3/14), and in the remaining groups, they occurred in a small percentage of patients.

**Figure 2:**
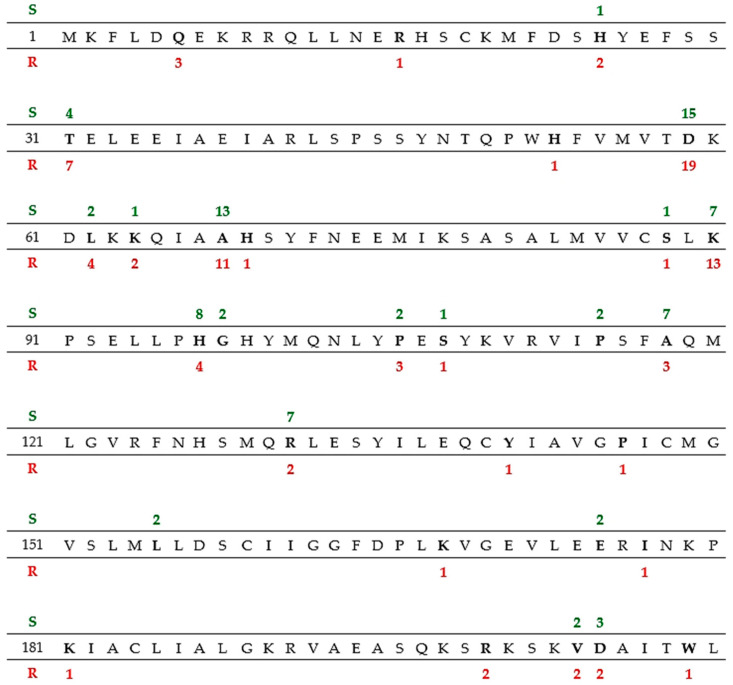
Sequence of the *rdxA* gene of *H. pylori* 26695 strain; green numbers above and red numbers below indicate the number of mutations detected in the tested strains that were sensitive (S) and resistant (R) to MET, respectively.

For the sequencing of the *rdxA* gene, 19 *H. pylori* strains resistant to MET and 16 strains sensitive to MET were selected. The complete amino acid sequence of the *rdxA* gene is presented in Figure 2. Point mutations were present at 166 loci of the gene. In tested MET-resistant strains, point mutations were identified at the following positions: three at position 6, one at 16; two at 25; seven at 31; one at 53; nineteen at 59; four at 62; two at 64; eleven at 68; one at 69 and 88; thirteen at 90; four at 97; three at 106; one at 108; three at 118; two at 131; one at 141, 146, 168, 177 and 181; two at 200, 204 and 205; and one at 209. In strains sensitive to MET, the following mutations were identified: one at position 25, four at 31, fifteen at 59, two at 62, one at 64; thirteen at 68, one at 88, seven at 90, eight at 97, two at 98 and 106, one at 108, two at 115, seven at 118, seven at 131, two at 174 and 204 and three at 205. Many point mutations were found in both MET-sensitive and MET-resistant strains (387 vs. 400, respectively; Table 3). The difference in the number of mutations between sensitive and resistant strains was statistically significant (*p* = 0.03, χ^2^ = 4.3909). Mutations that caused amino acid changes occurred with the same frequency among resistant and sensitive strains, while the main difference in the number of mutations was due to variability in regulatory regions of the gene. Resistant strains were more variable within the analyzed gene. Moreover, among resistant strains, stop codons formed as a result of mutations four times more often, which subsequently led to protein inactivation (Table 4). The occurrence of point mutations did not depend on the clinical diagnosis of the children from whom the strains were isolated.

Children from whom sensitive strains were isolated were treated for 7–14 days with standard eradication regimens according to the ESPGHAN/NASPGHAN guidelines [8], whereas children infected with strains resistant to CH or MET or double-resistant strains were treated with sequential therapy. The eradication efficacy was assessed with the rapid urease test (RUT) or endoscopy if necessary. The eradication efficacy was 100% for sequential therapy for CH-resistant strains and 72–75% for MET-resistant or double-resistant strains (data not shown).

## 4. Discussion

The incidence of *H. pylori* infection in children, despite better knowledge of its pathogenesis and therapy regimens, is still high (approximately 33%), which was demonstrated in two meta-analyses [18]. In Poland, the incidence of infection is similar, counting for approximately 30% [19]. The therapy regimens based on PPIs and two antibiotics (MET or CH and AMO) are used according to the ESPGHAN/NASPGHAN guidelines. The use of CH should be limited to cases where the sensitivity to this antibiotic has been confirmed, however, due to global increases in antibiotic resistance, eradication rates are unsatisfactorily low [8,20]. A recent multicenter meta-analysis involving 17 pediatric centers from 14 European countries demonstrated that resistance to CH reached 24%, resistance to MET was equal to 25%, resistance to both antibiotics (CH + MET) reached 7%, whereas resistance to amoxicillin was low and did not exceed 1% [20]. Resistance to CH shows marked differences depending on the geographical region and is caused by the high consumption of this antibiotic in children, mainly due to respiratory tract infections [1,8]. Our study shows that in our region single resistance to CH has doubled from 17.1% in the period from 2008–2012 to 31% in the current study, as was demonstrated in previous observations in a similar group of patients [13]. Analogous findings were observed by Shu et al. who showed a significant increase in CH resistance from 11% in 2012 to 26% in 2014 [21]. Similar increasing trends in CH resistance in children was observed by other authors from Europe and Asia [22,23,24]. The single resistance of *H. pylori* strains isolated from children to MET in our region remains stable, but high (from 28.6% in the period from 2008–2012 to current 31%), similar to other European countries (15–30%), but it is much higher in Iran (60%) and in China (68%) [13,20,21,22,25,26,27,28]. The increase in dual resistance to CH and MET from 14% to 35% detected in our study is particularly alarming. Shu et al. recently showed a significant increase in resistance rates for CH, MET and double-resistance to CH and MET in children in China [21]. Similar observations were made by researchers from Turkey [29]. In Poland, due to high resistance to CH (>15%), the empirical use of PPIs, MET and AMO in first-line therapy is a standard treatment, although at our center, attempts at targeted treatment based on results of susceptibility testing have been undertaken for several years [30]. Multiresistant strains, including those resistant to CH and MET, were detected significantly more frequently in boys with peptic ulcer disease and chronic gastritis than in girls (*p* < 0.05). Sex-related differences in prevalence and resistance of *H. pylori* were reported in the literature [20]. These differences may be explained by exposure to antibiotics and health habits in boys and girls. Antibiotics which are used for other indications, mainly respiratory tract infections, more frequently in boys, could contribute to cure rate of *H. pylori* infection also. It seems that the occurrence of dual resistance carries a high risk of first-line therapy failure, and it is necessary to implement methods of susceptibility testing before treatment. Due to the limited use of *H. pylori* culture methods, which are frequently unavailable, and time consuming, molecular methods of drug susceptibility testing seem to be a promising alternative.

Our study showed important differences in detected point mutations among strains isolated from children with different diseases, although they were not statistically significant, which may have been due to the small number of patients in all examined groups. The most frequent mutation among strains resistant to CH was A2143G, which was detected more often among resistant than sensitive strains (44% vs. 7%). Strains with this mutation were isolated most frequently in all groups of patients; moreover, this mutation was dominant in children with gastric/duodenal ulcer disease. The occurrence of the A2143G mutation among strains isolated in Poland was also confirmed by other authors [31]. Additionally, this mutation seems to be dominant among *H. pylori* strains worldwide [32,33,34,35]. These findings have important clinical implications. Francavilla et al. demonstrated that the eradication rate of strains with the A2143G mutation was very low, reaching approximately 50%; hence, the presence of this mutation carries a higher risk of treatment failure [36], while the eradication rate of strains with A2142G mutation reaches approximately 80%. In our study, strains resistant to CH with the A2142G mutation were most often present in patients with GERD (27%, 4/15). This is an important finding, because according to the guidelines, all children with *H. pylori* infection and GERD who require long-term PPI treatment should achieve eradication [1]. It was shown that for strains with the A2142G mutation, the eradication rates reached 80%. These strains, as shown by our previous observations and by researchers from Asia, had lower MIC values than A2143G strains [16,33,34]. Strains sensitive to CH with the A2143G or T2182C mutation were very rare or not detected. The cause of this phenomenon is unknown. The T2182C mutation is responsible for low level of resistance to clarithromycin. It can be assumed that the T2182C mutation does not have a great importance in the resistance mechanism. Hence, it is possible another independent resistance mechanism is necessary for the presence of phenotypic resistance. Moreover, considering the presence of the A2143G mutation in these strains, the existence of another mechanism is possible, which only in combination with A2143G mutation, gives a resistance phenotype. Another explanation may be that isolated strains can consist of mixed bacterial population in the gastric specimens, i.e., the specimens may have both susceptible and resistant strains [9,25]. We speculate that in children with peptic ulcer disease primary *H. pylori* infections may be caused by strains with a different drug susceptibility profile than in children with IBD and GERD. This phenomenon requires further study, especially since data are scarce in the literature. We did not find any mutations in the 23S rRNA gene in approximately 10% of *H. pylori* strains resistant to CH, which may suggest another cause of resistance. Apart from 23S rRNA mutations, the mechanism of resistance to CH may be related to the expression of efflux resistance-nodulation division (RND) pumps and the ability to form biofilms, which seem to play a role not only in the acquisition of CH resistance, but also to lead to the formation of multiresistant strains [9,37]. Moreover, it was demonstrated that *H. pylori* strains in which A2143G or A2142G mutations were identified were more frequently found in the structure of the biofilms than in plankton [38].

*H. pylori* resistance to MET is a complex phenomenon and is likely associated with the inactivation of the *rdxA* and/or *frxA* genes [9,12]. It is assumed that the main cause of *H. pylori* resistance is the mutation of the *rdxA* gene, whereas mutations in the *frxA* gene increase the degree of resistance [39,40]. According to some researchers, resistance to MET results from the deficiency or absence of one of the products of the *rdxA* and *frxA* genes or both simultaneously [40]. All changes were distributed throughout the genes, which was also seen in our study. Some single point mutations resulted in the formation of stop codons and were four times more common among MET-resistant strains than MET-sensitive strains. Some authors reported that up to 40% of *rdxA* point mutations cause the formation of stop codons, which consequently shortens the length of polypeptide, which can be quickly degraded [39]. In this study, mutations in the same positions of the *rdxA* gene occurred in MET-resistant and MET-sensitive strains. However, Tanih et al. observed the Glu27Val mutation was characteristic of MET-sensitive strains [41]. Apart from that, there were many mutations in MET-resistant strains that were already described by other authors, e.g., Arg115Ile, Arg16His, Ser111Leu and Arg16Cys [41,42]. The knowledge of RdxA protein structure enables the interpretation of mutations, which may cause the loss of NADPH reductase function [11]. Another cause of MET resistance may be outer efflux proteins (OEP) in the cell membrane, which may pump out various substances from the cell. The inhibition of OEP proteins (HP0605 and HP0971) in MET-resistant strains resulted in higher sensitivity to the drug [9]. Moreover, some researchers have suggested that several mutations in Fur proteins may influence the sensitivity to MET by affecting the balance of Fur proteins, resulting in the expression of genes controlling cellular redox potential and the elimination of active MET products [43]. Moreover, Binh et al. reported a new *rpsU* gene in *H. pylori* strains encoding the ribosomal protein 30S S21, which is probably associated with MET resistance [39].

Our study has some limitations. We did not analyze other resistance mechanisms such as the efflux pump genes and mutations in the *frxA* gene, which may also be relevant for resistance to MET. Moreover, the study did not analyze the eradication rates in the examined children because we were unable to collect follow-up information about the eradication regimen and its effectiveness in all children. The study was a single-center analysis, which is why the results cannot necessarily be extrapolated to other centers. However, to our knowledge, this is the first study in Poland to analyze mutations determining resistance to MET in a pediatric population and one of few studies in which the relationship between the occurrence of certain mutations in *H. pylori* strains and the type of gastrointestinal disease has been examined in children.

## 5. Conclusions

In conclusion, this study showed an increasing trend in the presence of CH resistance and the occurrence of multiresistant *H. pylori* strains in children with primary infections, while resistance rates to MET were stable. We showed significant differences in detected point mutations related to resistance to CH among strains isolated from children with different gastrointestinal diseases. The most common mutation associated with resistance to CH was A2143G, which was dominant among *H. pylori* strains isolated from children with peptic/duodenal ulcer disease (80%). Mutations in the *rdxA* gene occurred significantly more frequently among MET-resistant strains than MET-sensitive strains, and the difference in the number of mutations resulted from variability in regulatory regions of the *rdxA* gene.

## Figures and Tables

**Table 1:** Characteristics of the examined group of children and drug sensitivity of *H. pylori* strains.

*H. pylori* Strains	All Patients [Total] (*n* = 91)		Chronic Gastritis (*n* = 35)		GERD (*n* = 14)		Gastric/Duodenal Ulcer (*n* = 15)		Other * (*n* = 27)	
	Male	Female	Male	Female	Male	Female	Male	Female	Male	Female
**Susceptible to CH**	17 (18.7%)	12 (13.2%)	7 (7.7%)	5 (5.5%)	3 (3.3%)	1 (1.1%)	1 (1.1%)	0 (0.0%)	4 (4.4%)	8 (8.8%)
**Resistant to CH**	25 (27.5%)	37 (40.7%)	13 (14.3%)	10 (11%)	4 (4.4%)	6 (6.6%)	6 (6.6%)	8 (8.8%)	5 (5.5%)	10 (11%)
	*p* = 0.032		*p* > 0.05		*p* = 0.01		*p* = 0.03		*p* > 0.05	
**Susceptible to** **CH + MET**	17 (18.7%)	40 (44%)	7 (7.7%)	13 (14.3%)	3 (3.3%)	7 (7.7%)	1 (1.1%)	8 (8.8%)	4 (4.4%)	18 (19.8%)
**Resistant to** **CH + MET**	25 (27.5%)	9 (9.9%)	13 (14.3%)	2 (2.2%)	4 (4.4%)	0 (0%)	6 (6.6%)	0 (0%)	5 (5.5%)	0 (0%)
	*p* > 0.05		*p* < 0.01		*p* < 0.01		*p* < 0.01		*p* < 0.01	
**Susceptible to CH + MET + LEV**	42 (46.1%)	47 (51.6%)	20 (22%)	13 (14.3%)	7 (7.7%)	7 (7.7%)	7 (7.7%)	8 (8.8%)	9 (9.9%)	18 (19.8%)
**Resistant to CH + MET + LEV**	0 (0%)	2 (2.2%)	0 (0%)	2 (2.2%)	0 (0%)	0 (0%)	0 (0%)	0 (0%)	0 (0%)	0 (0%)
	*p* < 0.01		*p* < 0.01		*p* > 0.05		*p* > 0.05		*p* > 0.05	

*n* = number, resistance to AMO and TET and resistance only to MET were not detected. Statistical analysis using multi-divisional tables included comparisons within groups (resistant-sensitive/male-female). * Other—celiac disease, IBD, iron deficiency anemia, growth retardation or allergies.

**Table 2:** The occurrence of point mutations in the 23S rRNA gene in *H. pylori* strains depending on the clinical diagnosis.

*H. pylori* Strains	Clinical Diagnosis				Significance
	Chronic Gastritis (*n* = 35)	GERD (*n* = 14)	Peptic/Duodenal Ulcer (*n* = 15)	Other * (*n* = 27)	
**R/without mutation**	11% (4)	7% (1)	0% (0)	4% (1)	*p* > 0.05
**R/A2142G**	8% (3)	21% (3)	7% (1)	0% (0)	
**R/A2143G**	40% (14)	36% (5)	80% (12)	44% (12)	
**R/T2182C**	6% (2)	7% (1)	7% (1)	7% (2)	
**S/without mutation**	23% (8)	21% (3)	7% (1)	33% (9)	
**S/A2143G**	3% (1)	7% (1)	0% (0)	4% (1)	
**S/T2182C**	9% (3)	0% (0)	0% (0)	7% (2)	

*n*—number; R—resistant; S—sensitive; *—Other celiac disease; IBD; iron deficiency anemia; growth retardation or allergies.

**Table 3:** Frequency of changes in *rdxA* gene sequences obtained from 16 metronidazole (MET)-sensitives and 19 MET-resistant *H. pylori* strains.

*H. pylori* Strains	Number of Mutations	Number of Analyzed Positions without Mutations	Significance
MET-sensitive *n* = 2656 (16 × 166)	387	2269	*p* = 0.0361 (Chi^2^ = 4.3909)
MET-resistant *n* = 3154 (19 × 166)	400	2754	

*n* = number of total analyzed positions with potential mutations; 16 and 19 stands for the number of MET sensitive and resistant strains, respectively; 166 stands for the number of loci of the gene.

**Table 4:** Occurrence of point mutations in the *rdxA* gene in tested *H. pylori* strains.

*H. pylori* Strains	All Patients	Mutations	Mutations per Patient	Stop Mutations	AA Changes *n* (%)	AA Changes per Patient
MET-sensitive	16	387	24.2	1	82 (21%)	5
MET-resistant	19	400	21	4	90 (22.5%)	4.7
